# Cord blood-endothelial colony forming cells are immunotolerated and participate at post-ischemic angiogenesis in an original dorsal chamber immunocompetent mouse model

**DOI:** 10.1186/s13287-020-01687-7

**Published:** 2020-05-07

**Authors:** Richard Proust, Anne-Charlotte Ponsen, Valérie Rouffiac, Chantal Schenowitz, Florent Montespan, Karine Ser-Le Roux, Frédéric De Leeuw, Corinne Laplace-Builhé, Philippe Mauduit, Edgardo D. Carosella, Sébastien Banzet, Jean-Jacques Lataillade, Nathalie Rouas-Freiss, Georges Uzan, Juliette Peltzer

**Affiliations:** 1 INSERM UMR-S-MD 1197/Ministry of the Armed Forces, Biomedical Research Institut of the Armed Forces (IRBA), Paul-Brousse Hospital Villejuif and CTSA Clamart, Clamart, France; 2 Paris-Saclay University, Paris-Sud University, Gustave Roussy Institute, INSERM, CNRS, Molecular Analysis, Modeling and Imaging of Cancer Disease, Villejuif, France; 3 CEA, DRF-IBFJ, Hemato-Immunology Research Unit, INSERM UMR-S 976, IRSL – Paris University, Saint-Louis Hospital, Paris, France

**Keywords:** Endothelial progenitor cells, Cord blood-endothelial colony forming cells, Immunotolerance, Ischemia, Angiogenesis

## Abstract

**Background:**

Cardiovascular diseases are the main cause of morbidity and mortality worldwide. Restoring blood supply to ischemic tissues is an essential goal for the successful treatment of these diseases. Growth factor or gene therapy efficacy remains controversial, but stem cell transplantation is emerging as an interesting approach to stimulate angiogenesis. Among the different stem cell populations, cord blood-endothelial progenitor cells (CB-EPCs) and more particularly cord blood-endothelial progenitor cell-derived endothelial colony forming cells (CB-ECFCs) have a great proliferative potential without exhibiting signs of senescence. Even if it was already described that CB-ECFCs were able to restore blood perfusion in hind-limb ischemia in an immunodeficient mouse model, until now, the immunogenic potential of allogenic CB-ECFCs remains controversial. Therefore, our objectives were to evaluate the immune tolerance potency of CB-ECFCs and their capacity to restore a functional vascular network under ischemic condition in immunocompetent mice.

**Methods:**

In vitro, the expression and secretion of immunoregulatory markers (HLA-G, IL-10, and TGF-β1) were evaluated on CB-ECFCs. Moreover, CB-ECFCs were co-cultured with activated peripheral blood mononuclear cells (PBMCs) for 6 days. PBMC proliferation was evaluated by [3H]-thymidine incorporation on the last 18 h. In vivo, CB-ECFCs were administered in the spleen and muscle of immunocompetent mice. Tissues were collected at day 14 after surgery. Finally, CB-ECFCs were injected intradermally in C57BL/6JRj mice close to ischemic macrovessel induced by thermal cauterization. Mice recovered until day 5 and were imaged, twice a week until day 30.

**Results:**

Firstly, we demonstrated that CB-ECFCs expressed HLA-G, IL-10, and TGF-β1 and secreted IL-10 and TGF-β1 and that they could display immunosuppressive properties in vitro. Secondly, we showed that CB-ECFCs could be tolerated until 14 days in immunocompetent mice. Thirdly, we revealed in an original ischemic model of dorsal chamber that CB-ECFCs were integrated in a new functional vascular network.

**Conclusion:**

These results open up new perspectives about using CB-ECFCs as an allogeneic cell therapy product and gives new impulse to the treatment of cardiovascular diseases.

## Background

Cardiovascular diseases are the main cause of morbidity and mortality worldwide [1–3]. Restoring blood supply to ischemic tissues is an essential goal for a successful treatment of these diseases [4]. Several therapies based on delivery of pro-angiogenic factors, like VEGF, HGF, bFGF, or HIF-1, were considered as attractive treatment options to restore vascular network within ischemic tissues [5, 6]. Growth factor therapy consists in delivering corresponding recombinant proteins by intra-arterial administration to stimulate angiogenesis. Although pre-clinical studies were promising, larger randomized clinical trials failed to demonstrate significant benefits [4]. This gene therapy is based on delivery of genes encoding angiogenic growth factors by intramuscular plasmid or viral vector injection. During the last two decades, many clinical trials assessed the efficacy and security of gene therapy products for critical limb ischemia. However, the effectiveness of gene therapy using pro-angiogenic factors remains controversial, and only further large-scale clinical trials will clarify their efficacy [7–9].

Recently, cell transplantation-based therapy using stem or progenitor cells from different sources into ischemic tissues emerged as a new approach to stimulate angiogenesis and/or vasculogenesis. Among the different cell populations used, endothelial progenitor cells (EPCs), mesenchymal stromal cells (MSCs), and bone marrow-mononuclear cells demonstrated their benefit in pre-clinical studies for ischemic disease treatment [10–12].

EPCs were discovered by Asahara in 1997 and include different cell progenitors with heterogeneous lineage origin and functions [13, 14]. In vitro, mainly two distinct cell types have been identified [15]: (i) early EPCs or colony forming unit-endothelial cells (CFU-ECs) with myeloid phenotype displaying, in vivo, a paracrine angiogenic effect [16] and (ii) late EPCs or endothelial colony forming cells (ECFCs), also known as outgrowth endothelial cells that are the only progenitors with the ability to migrate to ischemic site and directly incorporate into new vessels during vascular network formation in vivo [14, 17, 18]. ECFCs could be isolated from different sources: bone marrow [19, 20], adult peripheral blood (APB) [13] or embryonic annexes like the umbilical cord blood [14, 17], umbilical cord [21], or more recently term placenta [22, 23].

Autologous APB-ECFCs appeared as the ideal candidates for cell therapy because of no risk of immunological rejection. However, when isolated from patients with vascular diseases, their low number and poor expansion properties limit their use as a cell therapy product.

Comparative studies demonstrated that CB-ECFCs are more clonogenic and have a greater proliferative potential without exhibiting signs of senescence [14, 18]. Moreover, we demonstrated that the immaturity, more preserved in CB-ECFCs than in APB-ECFCs, is an essential prerequisite for vascular repair functionality [24]. Indeed, Au et al. demonstrated that in vivo co-implantation of CB-ECFCs together with pericytes in collagen gel implanted into cranial windows in SCID mice led to long-lasting and functional vascular network, while APB-ECFC-derived blood vessels were transient and almost completely disappeared in 21 days [17]. Finally, we and others have demonstrated that CB-ECFCs restore blood perfusion in hind-limb ischemia performed in an immunodeficient mouse model [25, 26]. For all these reasons, despite a potential alloantigenic reaction from the host, CB-ECFCs seem to be the best valuable therapeutic tool. However, the non-immunogenic potential of allogeneic CB-ECFCs remains controversial. Some studies reported that CB-ECFCs display low immunogenic features [27, 28]. Finally, we have demonstrated in vitro that human CB-ECFCs could exert an immunosuppressive effect on mouse lymphocyte proliferation more accentuated than APB-ECFCs. Others stated that this low immunogenic state of ECFCs could only be obtained by co-culture with mesenchymal stromal cells [2, 3, 29]. To our knowledge, only Teofili’s group used CB-ECFCs in a hind-limb ischemia model in immunocompetent mice, suggesting the immune tolerance of CB-ECFCs. These authors demonstrated that human CB-ECFCs improve blood flow recovery and restrain the damaging ischemia effect. However, the physical incorporation of human CB-ECFCs in the mouse vascular network was not clearly demonstrated [30].

Given the therapeutic interest of CB-ECFCs and the limited literature data concerning their immunogenicity, our study aims to verify their persistence in an allogeneic environment and to follow over time their ability to participate in the repair of a vascular network in an innovative model in immunocompetent animals.

In the present study, we demonstrated that CB-ECFCs express immunoregulatory markers like HLA-G, IL-10, and TGF-β1, which are hypoimmunogenic and display immunosuppressive properties in vitro in a mixed lymphocyte reaction assay. We confirmed in vivo these tolerogenic features since human CB-ECFCs were still present 14 days after injection in the spleen and muscle of immunocompetent mice. Finally, using an original model of a dorsal chamber model implanted in immunocompetent mice, we proved that CB-ECFCs were not only tolerated but are also functional, since they directly integrated in a new functional vascular network under ischemic conditions.

## Methods

### Endothelial cell (EC) isolation and culture

Human umbilical cord blood samples were collected from healthy full-term newborns after signed maternal informed consent. According to the French law, article L.1243-3 of the Public Health Code, prior approval by an Institutional Review Board was not required. Samples were obtained through a partnership with the Cord Blood Bank of St Louis Hospital which is authorized by the National Agency for the Safety of Medicines and Health Products (ANSM; authorization no. PPC51). CB-ECFCs were isolated from cord blood mononuclear cells (CBMCs) collected by Ficoll (PAN-Biotech, Dutscher) density gradient centrifugation and were resuspended in a EGM-2-MV medium (Lonza, Levallois) as previously described [31, 32]. Briefly, CBMCs were then seeded in 12-well culture plates coated with 50 μg/mL rat tail collagen type I (Corning, Boulogne-Billancourt) and cultured at 37 °C, under 5% CO_2_, in a humidified incubator. After 24 h of culture, non-adherent cells and debris were aspirated. Adherent cells were washed once with PBS, and complete EGM-2-MV medium was added to each well. The medium was changed daily for 7 days and then every 2 days until the first passage. Colonies of endothelial cells appeared between 7 and 14 days of culture and were identified as well-circumscribed monolayers of cells with a cobblestone appearance. For all the experiments, CB-ECFCs were used at passages 3–5.

### Flow cytometry

CB-ECFCs were detached with trypsin and immunolabeled with BD Cytofix/Cytoperm™ Fixation/Permeabilization kit according to manufacturer’s protocol (BD Biosciences). Briefly, CB-ECFCs were fixed and permeabilized in Fixation/Permeabilization solution for 20 min at 4 °C. After 2 washings in BD Perm/Wash buffer, CB-ECFCs were immunolabeled with anti-soluble HLA-G5 (clone 2A12; Thermo Fisher Scientific), anti-IL-10 (clone B-S10; Diaclone), or anti-TGF-β1 (clone #27235; R&D Systems) antibodies for 30 min at 4 °C. After 2 washings in BD Perm/Wash buffer, secondary antibody GAM-PE was incubated for 30 min at 4 °C. After 2 washings in BD Perm/Wash buffer, data were acquired and analyzed on an Accuri C6 flow cytometer (BD Biosciences).

### CB-ECFC immunofluorescence staining

CB-ECFCs were grown in coverslips, fixed in 4% paraformaldehyde for 10 min at RT, rinsed twice in phosphate-buffered saline (PBS), and permeabilized in PBS/BSA 3%/Triton 0.1% for 5 min at RT. Soluble HLA-G5 (clone 2A12), IL-10 (clone B-S10), TGF-β1 (clone # 27235), and CD31 immunostaining were performed for 1 h at RT. After 3 washings in PBS/BSA 3%/Triton 0.1%, CB-ECFCs were incubated with secondary antibodies goat anti-rabbit-Alexa Fluor 488 (Invitrogen) and donkey anti-mouse-Alexa Fluor 568 (Invitrogen) for 45 min at RT. After 3 washings in PBS/BSA 3%/Triton 0.1%, CB-ECFCs were then stained with 2 μg/mL of 40,6-diamidino-2-phenylindole (DAPI) for 5 min at RT. After 3 washings in PBS 1×, slides were embedded using Glycergel Mounting Medium (Dako). Images were acquired with the Leica SP5 confocal microscope (Leica Microsystems, Wetzlar, Germany) equipped with a × 63 oil immersion fluorescence objective.

### ELISAs

CB-ECFCs were grown for 24 h in EBM-2 supplemented with 5% FBS. ELISAs against IL-10 and TGF-β1 (R&D Systems) were performed on supernatants according to manufacturer’s instructions.

### Mixed lymphocyte reaction (MLR)

CB-ECFCs were used as either stimulator (immunogenicity assay) or third-party cells (immunosuppression assay) toward HLA-mismatched peripheral blood mononuclear cells (PBMCs), as responder cells. PBMCs were isolated from the blood of healthy volunteer donors from the French Blood Establishment (EFS, Saint-Louis Hospital, Paris, France) after informed consent, by density gradient centrifugation over Ficoll-Paque PLUS (Sigma). Human B lymphoblastoid cell line (LCL) 721.221 (ATCC), HLA class II-positive cells, was irradiated at 75-Gy dose to be used as a stimulator in immunosuppression assay. The ratio of PBMCs and LCL seeded in each well of 96-well plate was 1:0.5, with a final concentration of 10^5^ PBMCs/well. CB-ECFCs were used at various ratios (from 0.5 × 10^5^ to 0.03 × 10^5^ cells/well). MLR lasted for 6 days at 37 °C in a humidified 5% CO_2_ air atmosphere. On day 5, [3H]-thymidine (1 mCi/well, PerkinElmer) was added to each well and incubated for another 18 h. Cells were then harvested on filtermats A, and thymidine incorporation into DNA was quantified using a β counter (Wallac 1450; Pharmacia).

### CB-ECFC transduction

At day 0, CB-ECFCs were incubated at 10,000 cells/cm^2^ in EGM-2-MV medium (Lonza). At day 1, CB-ECFCs were infected with lentiviral vector containing the GFP (rLV.EF1.GFP, Vectalys) or mCherry (pEZ-Lv105, GeneCopoeia) transgene with 8 μg/mL polybrene (SIGMA-Aldrich) at a multiplicity of infection (MOI) of 5 for 24 h at 37 °C. After washing with PBS, CB-ECFCs were amplified and transduction efficiency was evaluated by flow cytometry analysis (Accuri).

### Animal

All experimental procedures were performed in accordance with the European Community Council Directive (2010/63/UE) for the care and use of laboratory animals. Procedures on animals were authorized by the Ministère de l’Education Nationale, de l’Enseignement Supérieur et de la Recherche after approbation by the National Committee for Ethics in Animal Experimentation (CEEA N°26; project 2017030813196126_v2).

### CB-ECFCs-GFP^+^ intrasplenic and intramuscular administration

After anesthesia by intraperitoneal injection of ketamine (100 mg/kg)/xylasine (20 mg/kg), eight-week-old male C57BL/6JRj mice were incised on their left lateral side to access to the spleen. Cells were injected intrasplenically into a single point. After wound suturing, a second injection of the same treatment was performed intramuscularly in the quadriceps. Sixteen C57BL/6JRj mice were divided into 2 groups (Gn): (G1)—8 control mice were injected with 40 μL of PBS per injection, and (G2)—8 treated mice were injected with 1.10^6^ CB-ECFCs-GFP^+^ suspended in 40 μL of PBS per injection. The spleen and quadriceps were collected at day 14 after surgery, embedded in Tissue-Tek OCT compound, frozen in liquid nitrogen, and kept at − 80 °C.

### Muscle and spleen immunofluorescence staining

Frozen samples (muscle and spleen) were sectioned to 8 μm at − 21 °C using cryostat (Leica). Sections of the muscle and spleen were fixed in 4% paraformaldehyde for 10 min at RT and rinsed three times in PBS, and non-specific sites were saturated with PBS/NGS 5% for 1 h at RT. Anti-human-CD31 immunostaining was performed for 1 h at RT. After 3 washings in PBS/NGS 5%, muscle and spleen sections were incubated with secondary antibodies goat anti-mouse-Alexa Fluor 546 (Invitrogen) for 1 h at RT. After 3 washings in PBS/NGS 5%, sections were then stained with 2 μg/mL of DAPI for 5 min at RT. After 3 washings in PBS, slides were embedded using Glycergel Mounting Medium (Dako). Images were acquired with a Leica SP5 confocal microscope (Leica Microsystems, Wetzlar, Germany) equipped with × 63 oil immersion fluorescence objective.

### Dorsal chamber implantation and CB-ECFCs-mCherry^+^ injection

Nine-week-old male C57BL/6JRj mice were weighed before anesthesia by gas inhalation (1.5% isoflurane in 1.5–2 L/min air flow). The surgical procedure was previously described in details [33]. Briefly, each mouse received a subcutaneous injection of 100 μL of Lidocaïne® (21.33 mg/mL) in the scapular region before skin incision. A local antiseptic solution (Betadine®) was also applied. A skinfold was stretched keeping the dorsal median line at the top. The two faces of the dorsal chamber were positioned on each side of the skinfold and secured by sutures between two adjacent orifices. To limit the inflammatory reaction, a dermocorticoid ointment was spread on the side where the skin had not been removed. On the opposite side, the entire epidermis and upper dermis within the optical window was removed as close as possible to the edges. Cells were injected intradermally close to ischemia induced by thermal cauterization of a macrovessel 15 min before cell injection to allow sufficient heat dissipation for preventing cell injuries. Twelve C57BL/6JRj mice were included and divided into 3 groups (Gn): (G1)—4 control mice injected with 20 μL of PBS with vascular ischemia, (G2)—4 treated mice injected with 500,000 CB-ECFCs-mCherry^+^ suspended in 20 μL of PBS with ischemia, and (G3)—4 treated mice injected with 500,000 CB-ECFCs-mCherry^+^ suspended in 20 μL of PBS without ischemia. The surgical procedure was ended by closing the dorsal chamber with glass coverslips maintained by rings sealed by mechanical pressure. Anesthetic gas was switched off, and Tolfedine (4 mg/kg) was subcutaneously injected before the animal woke. Mice recovered inside cages enriched with a cocoon and diet gel to facilitate food access while the animals regained their initial mobility. Mice were maintained for a maximum of 30 days after dorsal chamber implantation.

### Imaging procedure

Mice recovered until day 5 and were imaged until day 30 twice a week onto a macroscope (AZ100M, Nikon) and once a week onto a confocal microscope (SP8, Leica) in wildfield and fluorescence mode after 100 μL dextran 2000 kDa-FITC intravenous injection (25 μg/μL).

For macroscopic assessment, mice were placed in a lateral position on a heating table maintained at 37 °C, itself placed on the macroscope stage. Acquisitions were recorded with a × 1 objective (numerical aperture (NA), 0.1) with 1.3 zoom which provided an image of the whole dorsal chamber in a single frame in bright field and fluorescence. Dextran 2000-kDa-FITC signal was detected using a GFP filter (emission (em), 515–550 nm bandwidth), while CB-ECFCs-mCherry^+^ were observed with a TexasRed filter (em, 573–613 nm bandwidth).

Following macroscopic imaging, mice were examined in confocal microscopy. To prevent mice from developing hyperthermia and dehydration during extended acquisitions, the confocal microscope was enclosed in an incubator (OkoLab®) connected to a control unit monitoring air flow, heating, and humidity. The dorsal chamber was mechanically locked unto a dedicated support adapted to the microscope stage, thus preventing image degradation due to cardiac and respiratory movements. Confocal acquisitions were performed with a × 10 (N.A. 0.40, air objective) and × 25 (N.A. 0.95, water objective) objective lens. Scanning speed was set at 600 Hz using the bi-directional acquisition mode, for a 1024 × 1024 pixel or image and a Z-step between 8 and 12 μm. A mosaicking technique was required to scan CB-ECFCs and ischemia on a same image. Moreover, a slight frame average of 2 was applied to deblur image. The 488-nm and 552-nm laser powers for the green and red illumination were set at 5% of their maximal power in most cases (25 mW and 20 mW respectively) and collected at 495–545 nm and at 562–622 nm respectively. We typically used these settings, but they could be adapted depending on fluorescent levels recorded at each time point.

### Statistical analysis

The allostimulatory effect of CB-ECFCs on T cells was evaluated by comparing the mean of each donor’s maximal stimulatory effect to the value (100%) represented by the LCL (positive control) condition with the one-sample, one-sided Wilcoxon test. The inhibitory effect of CB-ECFCs as a third party cell was evaluated by comparing to 0 the slope of a linear regression of the PBMC proliferation relative to the no-CB-ECFC condition (100%) by the numeric level of the increasing CB-ECFC numbers (0 being no-CB-ECFC, 1 being 2500 CB-ECFC, and so on). Tests and graphical representations were carried out using *stats*, *lmer*, *lmerTest*, and *ggplot2*packages in R (3.4.1). Results are described as mean and bootstrapped 95% confidence interval.

IL-10 and TGF-β1 quantifications were expressed as mean ± SD. Prism 7.0 (GraphPad Software) was used to perform the Mann-Whitney test for evaluating the difference in cytokine concentration in supernatants. A two-tailed *p* value < 0.05 was considered significant.

## Results

### CB-ECFCs are hypoimmunogenic and exert immunosuppressive properties

CB-ECFCs are thought to have a large potential in therapies aimed at repairing vascular defects from various etiologies. In this context, our present work entailed assessing, from an immunological perspective, whether allogenic CB-ECFCs could be used without a risk of rejection instead of autologous CB-ECFCs. For this purpose, we evaluated both immunogenicity and immunosuppressive properties of CB-ECFCs in HLA-mismatched settings.

To assess the immunogenicity of CB-ECFCs, we studied their ability to be recognized as allogeneic stimulating cells by HLA-mismatched PBMCs. Highly stimulatory HLA class II+ lymphoblastoid cell line (LCL) was used as a PBMC proliferation inducing control. Results show that PBMC alloproliferation means are significantly lower with CB-ECFCs (PBMC alloproliferation mean of 7.75% [IC95 2.66–7.84]) compared to HLA class II+ LCL (PBMC alloproliferation mean of 100%) (*p < 0.05).

**Fig. 1:**
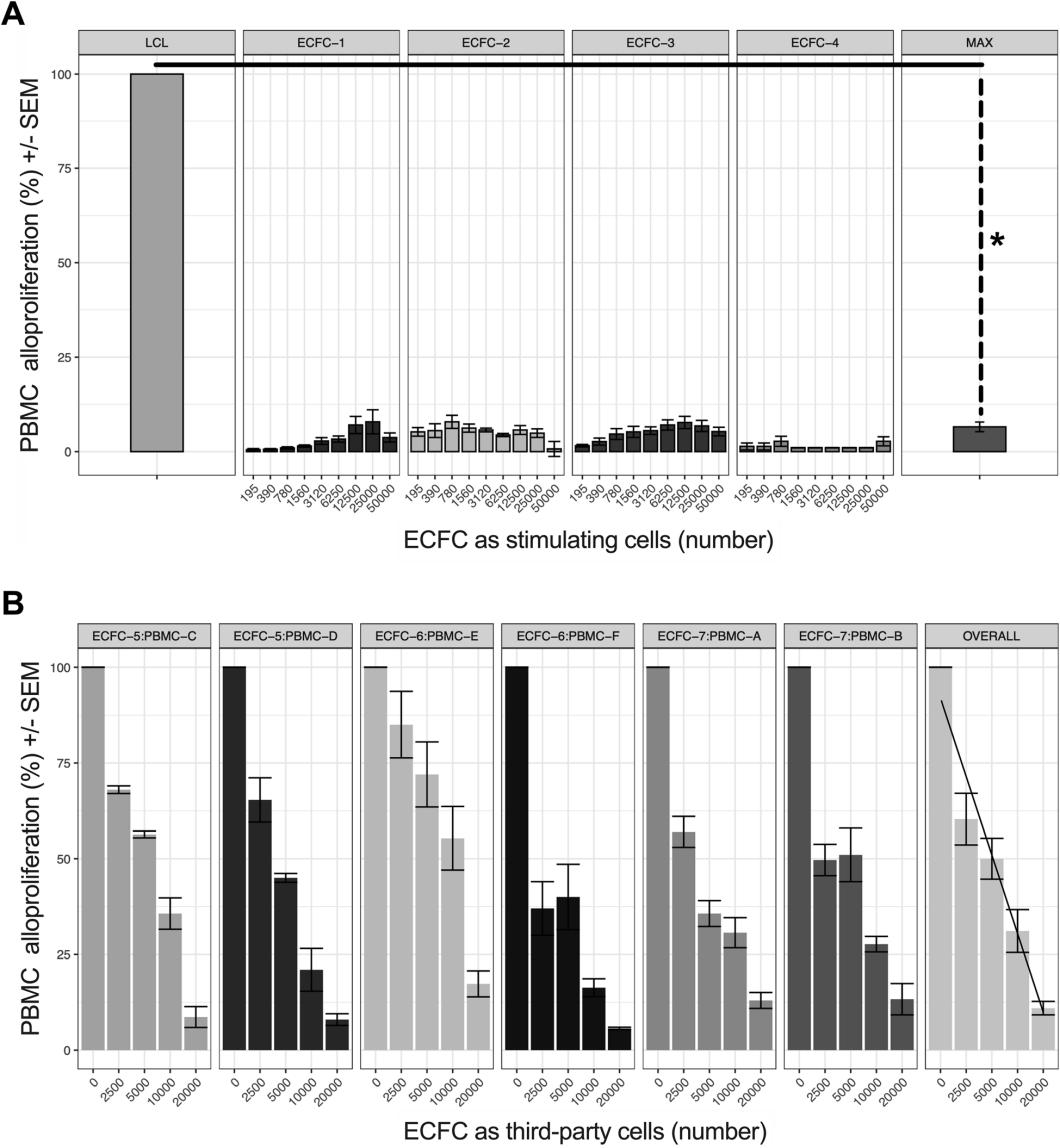
CB-ECFCs are hypoimmunogenic and exert immunosuppressive properties. **a** 10^5^ PBMCs were used as HLA-mismatched responder cells and stimulated by either various ratios of CB-ECFCs or by 0.5 × 10^5^ irradiated LCL cells as positive control. Data are given as histograms representing mean ± SEM of alloproliferation percentage obtained with 4 distinct PBMCs and 4 distinct CB-ECFCs (#1, #2, #3, #4); *p < 0.05. **b** 10^5^ PBMCs were used as HLA-mismatched responder cells, stimulated by 0.5 × 10^5^ irradiated LCL, and concomitantly inhibited by various ratios of CB-ECFCs as third-party cells. Data are represented as a linear dose effect of CB-ECFC number on alloproliferation percentage, obtained with 6 distinct PBMCs and 3 distinct CB-ECFCs (#5, #6, #7); the alpha angle represents the difference between the slope and 0

To examine the immunosuppressive features of CB-ECFCs, we studied their ability to affect PBMC alloproliferation as third-party cells in a classical MLR. Results revealed that CB-ECFCs significantly inhibit PBMC alloproliferation in a dose-dependent manner using 4 distinct PBMC to CB-ECFC allogeneic combinations (Fig. 1a). The slope of a linear regression of PBMC alloproliferation (relative to the no-CB-ECFC control), by CB-ECFC dose number, was significantly below 0 (*p < 0.05) (slope − 20.72 [IC95, − 21.62 to − 19.9]) indicating that CB-ECFCs exert a dose-dependent inhibitory effect on PBMC alloproliferation using 6 distinct PBMC to CB-ECFC allogenic combinations (Fig. 1b).

### CB-ECFCs express immunosuppressive markers HLA-G, IL-10, and TGF-β1

**Fig. 2:**
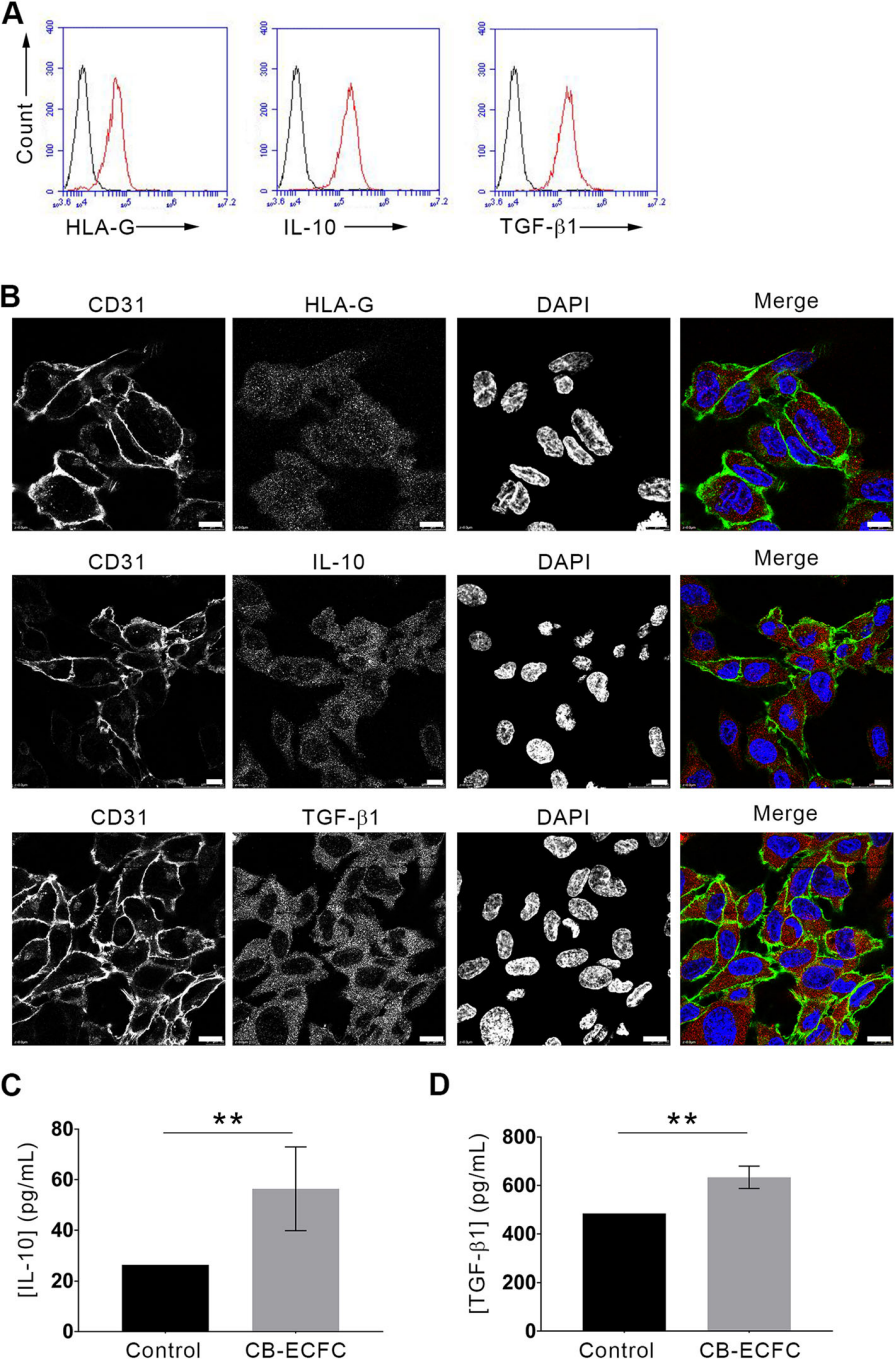
CB-ECFCs express immunosuppressive markers HLA-G, IL-10, and TGF-β1. **a** After fixation and permeabilization, CB-ECFCs were stained with anti-HLA-G5 (clone 2A12), anti-IL-10 (clone B-S10), and anti-TGF-β1 (clone #27235) antibodies. Protein expressions were monitored and analyzed on a BD Accuri™ C6 flow cytometer. Data are mean fluorescence intensities (MFI); representative histograms of 3 experiments are shown (black curves, IgG isotype control; red curves, markers). **b** CB-ECFCs were grown on glass coverslips, fixed, permeabilized, and stained (left panels, CD31 staining; middle left panels: HLA-G5 or IL-10 or TGF-β1 staining; middle right panels, DAPI staining; right panels, merges). Scale bars represent 10 μm. Representative images of 3 experiments are shown. **c**, **d** CB-ECFCs were grown for 24 h in EBM-2 supplemented with 5% FBS. Supernatants were collected and ELISAs were performed to quantify IL-10 (**c**) and TGF-β1 (**d**). Data are given as histograms representing means ± SD of 5 distinct CB-ECFCs; **p < 0.01; Mann-Whitney test

Some previous works evaluated the immunological potential of CB-ECFCs because of their protection against allospecific cellular immune response. However, the mechanisms that confer this protection (anti-inflammatory molecule secretion, cell-cell interaction, cytotoxicity …) are not well understood. In our team, we have recently demonstrated that the TNF/TNFR2 signaling pathway is a key regulatory factor in CB-ECFC immunosuppressive effect. In this study, we checked the expression of different anti-inflammatory cytokines by flow cytometry. As shown in Fig. 2a, CB-ECFCs constitutively expressed the anti-inflammatory cytokines IL-10 and TGF-β1 as well as the immune checkpoint HLA-G [34]. These results were confirmed by confocal microscopy. CB-ECFCs expressed IL-10, TGF-β1, and the soluble HLA-G5 isoform in small intracytoplasmic vesicles (Fig. 2b). Moreover, ELISA analysis of 5 distinct CB-ECFC supernatants showed that these cells were able to secrete IL-10 and TGF-β1 ([IL-10]_CB-ECFCs_ = 56.4 pg/mL and [TGF-β1]_CB-ECFCs_ = 633.7 pg/mL; **p < 0.01, Fig. 2c, d).

Weibel-Palade bodies (WPB) are large vesicles specific to ECs and serve as a stock for von Willebrand factor (vWF) and P-selectin. Thereby, ECs contribute to hemostasis and inflammation by release of WPB. However, using confocal microscopy, the anti-inflammatory cytokines IL-10, TGF-β, and HLA-G were not co-localized with vWF, suggesting that these cytokines are not stocked in WPB (data not shown).

### CB-ECFCs are tolerated in in vivo immunocompetent environment

To confirm the non-immunogenicity of CB-ECFCs in vivo, CB-ECFCs-GFP^+^ were injected in the muscle and spleen of immunocompetent mice.

**Fig. 3:**
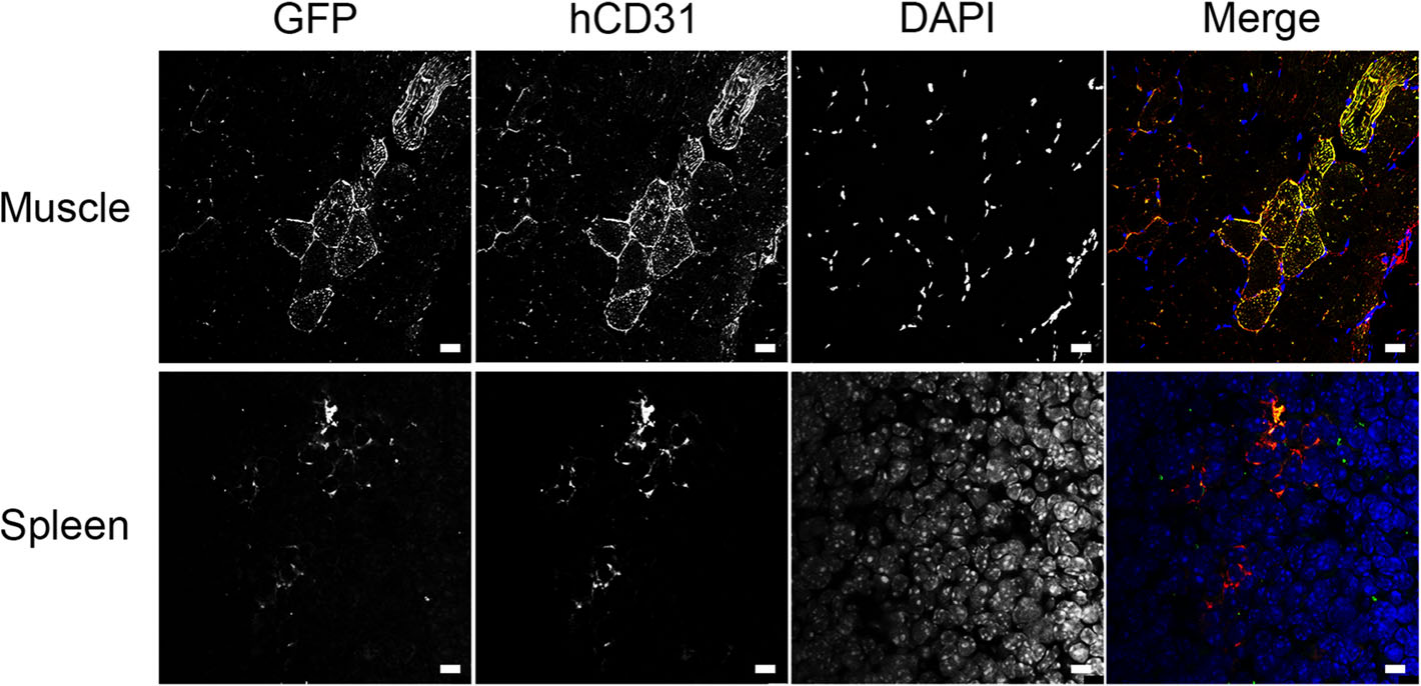
CB-ECFCs are tolerated after implantation in spleen and muscle immunocompetent mice. 1.10^6^ CB-ECFCs-GFP^+^ were injected into the spleen or muscle in C57BL/6JRj mice. Fourteen days after injection, spleens and muscles were collected and embedded in Tissue-Tek OCT compound. Cryosections of samples were stained (left panels, GFP staining; middle left panels: hCD31 staining; middle right panels, DAPI staining; right panels, merges). Scale bars represent 25 μm. Representative images of 8 mice are shown

D14 after transplantation, the presence of CB-ECFCs-GFP^+^ was observed in the muscle and spleen by confocal microscopy. This observation is confirmed by the co-localization of CB-ECFCs-GFP^+^ with specific human-CD31 immunostaining (Fig. 3).

### CB-ECFCs promote functional vascular network in immunocompetent mice

To study the functionality of CB-ECFCs in vivo, we used an original immunocompetent mouse model consisting of dorsal chamber with macrovessel ischemia. Compared to hind-limb ischemia, the advantage of this model was to directly visualize the evolution of the vascularization on the same living mouse over time.

**Fig. 4:**
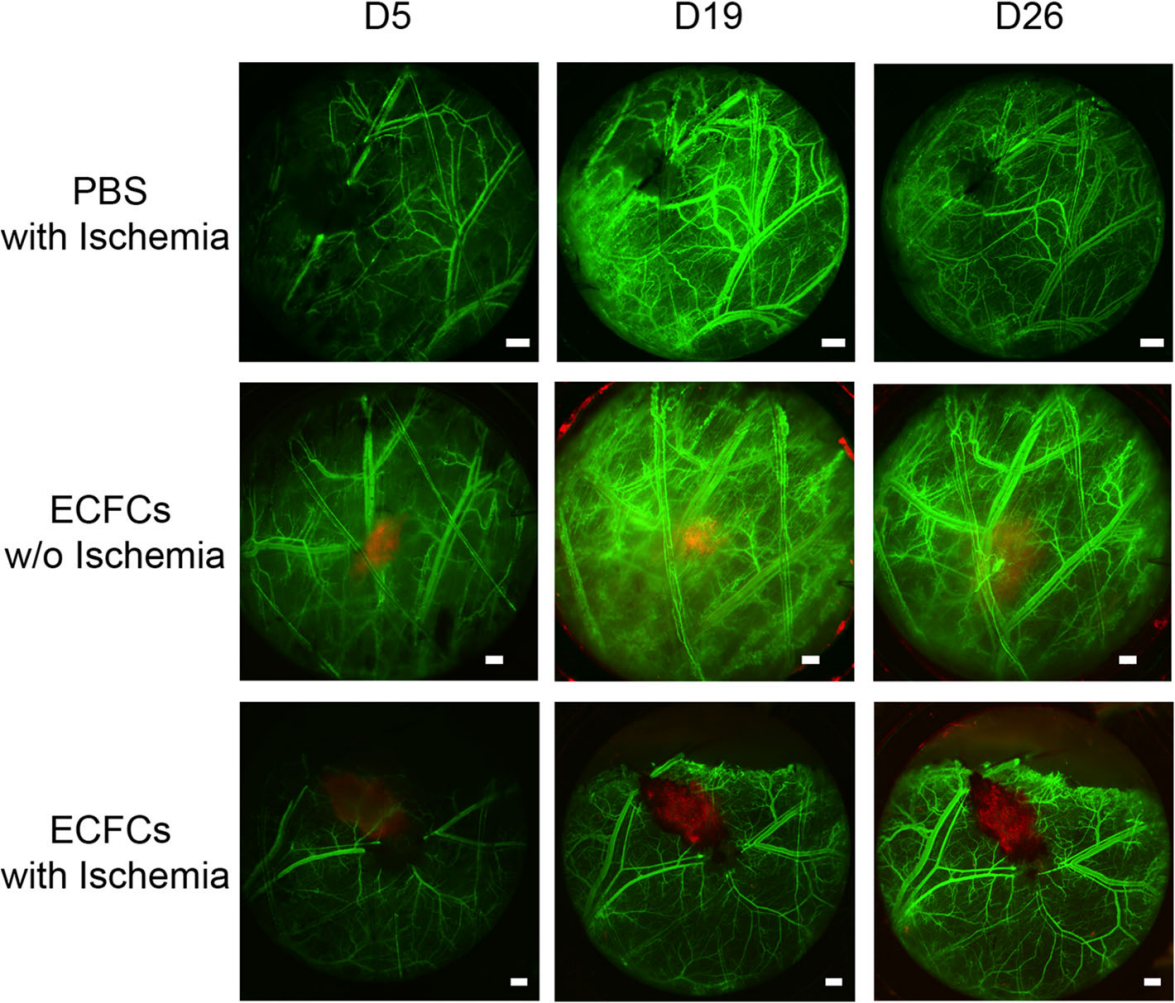
CB-ECFCs survive in ischemic environment. Fifteen minutes after ischemia (top and bottom panels) induced by thermal cauterization, PBS (top panels) or 5.10^5^ CB-ECFCs-mCherry^+^ (middle and bottom panels) were injected. Five, 19, and 26 days after treatment, dextran-FITC was injected by IV to reveal mouse vascular network. Scale bars represent 500 μm. Representative images of 4 mice per group are shown

Macroscopic analysis revealed that the fluorescence of CB-ECFCs-mCherry^+^ injected without macrovessel ischemia decreased over time and seemed to have almost disappeared at D26 (Fig. 4). This result was obtained using the same fluorescence exposure for the same mouse over time suggesting that the decrease in fluorescence over time, observed after injection of CB-ECFCs-mCherry^+^ without ischemia, corresponded to the death of these cells.

When CB-ECFCs-mCherry^+^ were injected in the ischemic environment, the fluorescence signal was maintained up to 26 days after injection (Fig. 4).

**Fig. 5:**
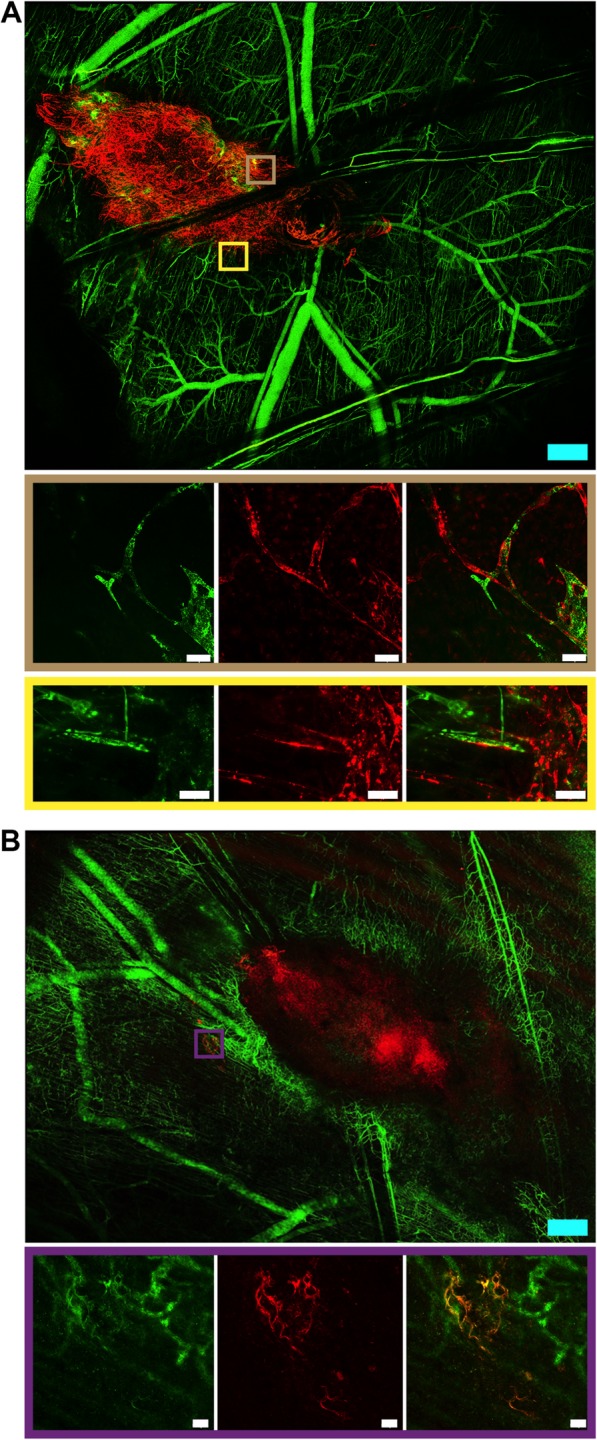
CB-ECFCs promote functional vascular network in immunocompetent mice. Twenty-six days after ischemia, CB-ECFCs-mCherry^+^ structured themselves into functional vessels, integrated mouse vascular network as revealed by dextran-FITC flow inside at the site of injection (**a**) or in periphery to the site of injection (**b**). Blue and white scale bars respectively represent 500 and 50 μm. Representative images of 4 mice per group are shown

When we focused in confocal microscopy on CB-ECFCs-mCherry^+^ with ischemia condition at D26, vessels lined with mCherry cells were observed within the cluster of human cells injected. Moreover, when dextran-FITC was injected into the mouse vascular circulation, real-time green fluorescent flow was observed in vessels lined with CB-ECFCs-mCherry^+^ (Fig. 5a), suggesting a connection between the mouse vascular circulation and human neo-vessels composed of human CB-ECFCs. More surprisingly, vessels lined with CB-ECFCs-mCherry^+^ were also founded at a distance from the human cell cluster (Fig. 5b).

## Discussion

In vascular diseases such as critical limb ischemia, restoring blood supply is essential to effectively repair damaged ischemic tissues. Animal ischemic models show that CB-ECFCs injected into ischemic lesion actively participate to the formation of new vessels, leading to durable healing [17, 35]. CB-ECFCs display high proliferative potential without obvious signs of senescence [24, 30]; it is thus possible to reach the needed therapeutic dose with still highly active cells. Therefore, recent works promote the use of CB-ECFCs as a promising allogenic source for cell therapy in ischemic diseases; however, their immunological properties in allogeneic therapeutic use remain unclear.

In our study, we demonstrate that CB-ECFCs are non-immunogenic and exert an immunosuppressive effect on PBMC response (HLA-mismatching). Indeed, we initially did not observe a significant alloproliferation of non-activated PBMCs, suggesting their low ability to be recognized as allogenic stimulating cells by HLA-mismatched PBMCs. In a second step, when CB-ECFCs were co-cultured with activated PBMCs, we observed a dose-dependent decrease in PBMC alloproliferation, suggesting an immunosuppressive capacity of these cells.

These results are in agreement with those obtained by Seifert and Pober groups who observed after stimulation with γ-IFN, a moderate proliferation of allogeneic CD4+ T cells cultured with EPC-derived ECs, demonstrating that these cells display a weak allostimulatory capacity against allogeneic CD4+ T cells in vitro [28, 36]. Control mature ECs from the same donor did not produce the same effect, indicating that this property is unique to EPC-derived ECs. Seifer et al. demonstrated in vivo that human CB-ECFCs transplanted in a xenogenic vascular graft model with decellularized aortic transplants did not trigger T cell infiltration and a weak infiltration of monocytes and macrophages suggesting that CB-ECFCs do not trigger any acute immune rejection. No intima remodeling was neither observed. In contrast, mature ECs triggered an immune response and massive inflammation [28]. To go further, our team demonstrated for the first time that the immunosuppressive effect of ECFCs is mediated by the TNF/TNFR2 signaling pathway.

Although several studies now tend to demonstrate that ECFCs display a certain level of non-immunogenicity, the underlying mechanisms are not yet fully understood. In this study, we hypothesized that the non-immunogenicity of CB-ECFCs could result from the expression of various anti-inflammatory molecules capable of protecting cells against the host’s immune system. Indeed, we have demonstrated that CB-ECFCs contain in their cytoplasm the soluble HLA-G5 isoform, as well as IL-10 and TGF-β molecules that are also be secreted by the cells.

Krishnamurthy et al. showed in a model of myocardial infarction in IL-10 KO mice treated or not with recombinant IL10 that EPC injected intramyocardially in the IL-10-treated group had a better survival. This was correlated with an improvement of angiogenesis and left ventricular function and the reduction of infarct size and fibrosis in the myocardium [37]. Moreover, the same team showed that IL-10 KO-EPC-derived exosomes inherit their parental dysfunctional phenotype. WT-EPC-Exo treatment enhanced endothelial cell proliferation and tube formation and inhibited apoptosis, whereas IL-10 KO-EPC-Exo exhibited impaired or even detrimental effects [38]. TGF-β also plays a key role in the angiogenic process of hypoxic tissue. For example, during stroke, neovascularization occurs primarily at the periphery of the infarct, which correlates with high levels of both mRNA and active TGF-β protein [39]. However, the expression levels could have contradictory effects. Indeed, low doses could promote angiogenesis and higher levels result in growth inhibition of endothelial cells and maturation of blood vessels [40, 41]. During immune injury, TGF-β inhibits leukocyte adhesion and transmigration via inhibition of IL-8 and E-selectin expression, decreases cytokine-stimulated inducible nitric oxide synthase production, and increases endothelial nitric oxide synthase expression. Therefore, several TGF-β actions on the endothelium during immune activation can be viewed as immunosuppressive [42]. Finally, HLA-G, a non-classical HLA class I molecule, initially detected at the maternal-fetal interface, exerts inhibitory functions on cells responsible for graft rejection, without triggering an allogeneic response. Data of literature also indicated that soluble HLA-G5 isoform act on T cell proliferation by inhibiting the progression of alloreactive T cells [43]. Our results are consistent with those obtained by the Rizzo team in 2011 demonstrating that CD34+ progenitor cells derived from the cord blood express different isoforms of HLA-G molecules [44]. Based on this study, Nuzzolo et al. also hypothesized that the cytotoxic response against ECFCs could be reduced by low expression of HLA molecules and/or expression of immunoregulatory molecules such as HLA-G. In their study, the authors demonstrated that ECFCs from adult peripheral and cord blood, express HLA-ABC (class I) and HLA-DR antigens (class II) at levels comparable to those of MSCs, both before and after γ-IFN stimulation. Allogenic MSCs are commonly administered in vivo and do not trigger immune rejection. In addition, they demonstrated that CB-ECFCs were able to decrease the expression of mediators of inflammation and atherosclerosis produced by allogeneic lymphocytes and monocytes (IL-1β, IL-8, PGF, ALOX-5, TNFα, CSF-2, MMP-1, MMP-9) more significantly than adult ECFCs [27].

In order to validate in vivo the non-immunogenic and immunosuppressive nature of CB-ECFCs, we first confirmed that these cells were not rejected after injection into the muscle and spleen of immunocompetent mice. Indeed, injected CB-ECFCs were still present 14 days after injection in both tissues. Then, we used an original model of ischemia in mouse using a dorsal chamber to demonstrate that beyond their tolerance by the immune system of the recipient mouse, CB-ECFCs are able to form new vessels 26 days after transplantation. This new model was recently described in the literature to study the tumoral angiogenesis [33]. We adjusted this model to directly visualize angiogenesis after ischemia in a living mouse. These results are consistent with those obtained by the team of Teofili in a model of hind-limb ischemia in the immunocompetent mouse. Despite a progressive decline in the number of hCD31^+^ cells over time, a significant amount of human cells were still detected 28 days after transplantation. In addition, they observed that mice treated with CB-ECFCs showed an increase in microvascular density as compared to control without CB-ECFCs. Human CD31^+^ cells were detected inside vessels up to 4 weeks post-injection, suggesting the development of microvasculature from human origin. In their study, Flex et al. also showed a weak intramuscular inflammatory infiltration after transplantation of CB-ECFCs similar to that observed in our current study [30].

In another study from Shafiee et al., the authors observed that when ECFCs were transplanted in immunodeficient mice, the efficacy of transplantation and the number of new vessels formed were higher than they were in immunocompetent mice, suggesting that the immune system is an important parameter for ECFC transplantation [2]. However, the ECFCs used in this study were isolated from the placenta and it is now clearly admitted that the origin of the progenitor cells, including the ECFCs, can deeply influence their immunogenic phenotype. For example, we have previously demonstrated that cord blood MSCs are better tolerated than MSCs derived from the umbilical cord Wharton jelly or from the placenta (amnion or chorion) [45]. We have also demonstrated that the immunosuppressive effect was more accentuated in CB-ECFCs as compared to APB-ECFCs.

We demonstrated for the first time in an immunocompetent mouse model of ischemia that the vessels formed by CB-ECFCs 26 days after transplantation are directly connected to the blood circulation of the mouse. Indeed, after injecting dextran-FITC into the bloodstream, we can detect a flow within the vessels formed by the CB-ECFCs. These results are in agreement with several studies performed in different models of immunodeficient mice demonstrating the vasculogenic potential of ECFCs, with the formation of blood vessels generally observed 3 weeks after cell injections and maintained for at least 6 weeks [17, 29, 35, 36, 46]. These results also appear to correlate with the Teofili study performed in a model of lower limb ischemia in immunocompetent mice where they observed a significant increase in blood perfusion in the ischemic limb 28 days after intramuscular CB-ECFC transplantation [30].

## Conclusion

Thus, through this study, we demonstrate for the first time that human CB-ECFCs, beyond their non-immunogenicity and immunosuppressive capacity, are able to form functional blood vessels in immunocompetent mice, directly connected to the endogenous vascular network.

This proof of concept opens up perspectives about using CB-ECFCs as an allogeneic cell therapy product. It represents an interesting breakthrough and gives new impulse to the treatment of ischemic diseases. Allogenic CB-ECFCs are readily available through cord blood banks, and the ethical issue for their use is already well defined. They offer a robust cellular source for promoting vascular repair within the framework of tissue engineering.

## Data Availability

All data generated or analyzed during this study are included in this published article with the exception of IL-10, TGF-β, HLA-G, and vWF co-localized study. This data are available from anne-charlotte.ponsen@inserm.fr.

## Abbreviations

ALOX-5: Arachidonate 5-lipoxygenase
APB: Adult peripheral blood
bFGF: Basic fibroblast growth factor
CB: Cord blood
CBMCs: Cord blood mononuclear cells
CD: Cluster differentiation
CFU-ECs: Colony forming unit-endothelial cells
CSF-2: Colony-stimulating factor-2
DAPI: 40,6-Diamidino-2-phenylindole
DNA: Deoxyribonucleic acid
ECFCs: Endothelial colony forming cells
EGM-2-MV: Endothelial growth media-2-microvascular
EPCs: Endothelial progenitor cells
FITC: Fluorescein isothiocyanate
GFP: Green fluorescent protein
Gn: Groups
HGF: Hepatocyte growth factor
HIF-1: Hypoxia-inducible factor 1
HLA: Human leukocyte antigen
IFN: Interferon
IL: Interleukin
LCL: Lymphoblastoid cell line
MLR: Mixed lymphocyte reaction
MMP: Matrix metalloproteinase
MOI: Multiplicity of infection
MSCs: Mesenchymal stromal cells
PBMCs: Peripheral blood mononuclear cells
PBS: Phosphate-buffered saline
PE: Phycoerythrine
PGF: Placental growth factor
RT: Room temperature
TGFβ: Transforming growth factor-β
TNFα: Tumor necrosis factor-α
VEGF: Vascular endothelial growth factor
vWF: von Willebrand factor
WPB: Weibel-Palade bodies
